# The Artificial Neural Networks Based on Scalarization Method for a Class of Bilevel Biobjective Programming Problem

**DOI:** 10.1155/2017/1853131

**Published:** 2017-09-14

**Authors:** Tao Zhang, Zhong Chen, June Liu, Xiong Li

**Affiliations:** ^1^School of Information and Mathematics, Yangtze University, Jingzhou 434023, China; ^2^School of Management, Huaibei Normal University, Huaibei 235000, China; ^3^School of Mathematical Sciences, Beijing Normal University, Beijing 100875, China

## Abstract

A two-stage artificial neural network (ANN) based on scalarization method is proposed for bilevel biobjective programming problem (BLBOP). The induced set of the BLBOP is firstly expressed as the set of minimal solutions of a biobjective optimization problem by using scalar approach, and then the whole efficient set of the BLBOP is derived by the proposed two-stage ANN for exploring the induced set. In order to illustrate the proposed method, seven numerical examples are tested and compared with results in the classical literature. Finally, a practical problem is solved by the proposed algorithm.

## 1. Introduction

The bilevel programming problem (BLP) is a nested optimizations problem with two levels in a hierarchy: the upper and lower level decision-makers. The upper level maker makes his decision firstly, followed by the lower level decision-maker. The objective function and constraint of the upper level problem not only rely on their own decision variables but also depend on the optimal solution of the lower level problem. The decision-maker at the lower level has to optimize his own objective function under the given parameters from the upper level decision-maker. Since many practical problems, such as engineering design, management, economic policy, and traffic problems, can be formulated as hierarchical problems, BLP has been studied and received increasing attention in the literatures. During the past decades, some surveys and bibliographic reviews were given by several authors [1–4]. Reference books on bilevel programming and related issues have emerged [5–8].

The bilevel programming problem is a nonconvex problem, which is extremely difficult to solve. As we know, BLP is a NP-Hard problem [9–11]. Vicente et al. [12] also showed that even the search for the local optima to the bilevel linear programming is NP-Hard. Even so, many researchers are devoted to develop the algorithms for solving BLP and propose many efficient algorithms. To date a few algorithms exist to solve BLP; it can be classified into four types: Karus-Kuhn-Tucker approach (KKT) [13–16], Branch-and-bound method [17], penalty function approach [18–21], and descent approach [22, 23].

Unfortunately, the bilevel programming problem is nonconvex and the properties such as differentiation and continuity are necessary when proposing the traditional algorithms. Thus, many researchers tend to propose the heuristic algorithms for solving BLP because of their key characteristics of minimal problem restrictions such as differentiation. Mathieu et al. [24] firstly developed a genetic algorithm (GA) for bilevel linear programming problem because of its good characteristics such as simplicity, minimal problem restrictions, global perspective, and implicit parallelism. Motivated by the same reason, other kinds of genetic algorithm for solving bilevel programming were also proposed in [25–28]. Because of the prominent advantage that neural computing can converge to the equilibrium point (optimal solution) rapidly, the neural network approach was used to solve bilevel programming problem in [29–31]. In additional, since McCulloch and Pitts [32] and Pyne [33] utilized logical calculus to emulate nervous activities, there have been various types of analogue neural networks proposed for computation. Sheng et al. [34] firstly proposed a neural network approach based on Frank-Wolfe method for a class of BLP problems. Shih et al. [35] and Lan et al. [29] presented neural network for solving linear BLP problem, respectively. Recently, Lv et al. [30, 36] investigated the nonlinear bilevel programming and the convex quadratic bilevel programming by neural network. However, there are few results on application of neural networks to the BLBMPP in the literature.

Particle swarm optimization (PSO) is a relatively novel heuristic algorithm inspired by the choreography of a bird flock. Due to its high speed of convergence and relative simplicity, the PSO algorithm has been employed for solving BLP problems. For example, Li et al. [37] proposed a hierarchical PSO for solving BLP problem. Kuo and Huang [38] applied the PSO algorithm for solving bilevel linear programming problem. Jiang et al. [39] presented the PSO based on CHKS smoothing function for solving nonlinear bilevel programming problem. Gao et al. [40] presented a method to solve bilevel pricing problems in supply chains using PSO. Zhang et al. [41] presented a new strategic bidding optimization technique which applies bilevel programming and swarm intelligence. The hybrid algorithms based on PSO are also proposed to solve the bilevel programming problems [42–44]. Besides, Tabu search [45–47], simulated annealing [48], ant colony optimization [49], and *λ*-cut and goal-programming-based algorithm [50] are also typical intelligent algorithms for solving bilevel programming problem.

However, the algorithms mentioned above are only for the simple single objective bilevel programming problems. In fact, the multiobjective characteristics widely existing in the BLPP and the bilevel multiobjective programming problem (BLMPP) have attracted many researchers' interesting. For example, Shi and Xia [51, 52], Abo-Sinna and Baky [53], and Nishizaki and Sakawa [54] presented an interactive algorithm for BLMPP. Eichfelder [55] developed a numerical method for solving nonlinear nonconvex bilevel multiobjective optimization problems. In recent years, the metaheuristic has attracted considerable attention as an alternative method for BLMPP. For example, Deb and Sinha [56–58] as well as Sinha and Deb [59] discussed BLMPP based on evolutionary multiobjective optimization principles. Based on those studies, Deb and Sinha [60] proposed a viable and hybrid evolutionary-local-search based algorithm and presented challenging test problems. Sinha [61] presented a progressively interactive evolutionary multiobjective optimization method for BLMPP. Lately, Zhang et al. [62] proposed an improved PSO for BLMPP and a framework of PSO for solving BLMPP is established. Subsequently, Zhang et al. [63] proposed an elite quantum behaved PSO for relatively complex BLMPP. In 2013, Zhang et al. [64] proposed a hybrid particle swarm optimization algorithm with crossover operator to solve high-dimensional BLMPP. Almost all the research object of the BLMPPs is the BLBOP, so we mainly consider the BLBOPs in this paper.

As we known, the authenticity of the lower level Pareto optimal solution is very important for the BLBOP. If the obtained optimal Pareto solutions possess the fraudulence, it can lead to the failure to solve the whole problem. In this paper, the induced set of the BLBOP is firstly expressed as the set of minimal solutions of a biobjective optimization problem by a scalar approach which can greatly improve the accuracy of the lower level Pareto optimal solutions. Based on the efficient set of the BLBOP, a two-stage ANN is presented for solving whole problem which can reduce the computation burden.

The remaining of this paper is organized as follows. In Section 2, we give the formulation of the model and related definitions. In Section 3, we will introduce the scalar approach for the induced set of the BLBOP and the two-stage ANN algorithm for the whole problem. In Section 4, some numerical examples and practical problem are given to demonstrate the feasibility and efficiency of the proposed algorithm, while the conclusion is reached in Section 5.

## 2. Problem Formulation and Main Theory Results

Let *X* be a nonempty subset of *R*^*n*_1_^, *Y* be a nonempty subset of *R*^*n*_2_^, and *F* : *R*^*n*_1_^ × *R*^*n*_2_^ → *R*^*m*_1_^, *f* : *R*^*n*_1_^ × *R*^*n*_2_^ → *R*^*m*_2_^, *G* : *R*^*n*_1_^ × *R*^*n*_2_^ → *R*^*p*^, and *g* : *R*^*n*_1_^ × *R*^*n*_2_^ → *R*^*q*^ be vector-valued mappings. We consider the following bilevel multiobjective programming problem (BLMPP):

(1) minx,y Fx,ys.t. x∈I,miny fx,ys.t. gx,y≥0,x∈X,  y∈Y,

where *F*(*x*, *y*) and *f*(*x*, *y*) are the upper level and the lower level objective functions, respectively. *x* ∈ *I*⊆*X* and *g*(*x*, *y*) denote the upper level and the lower level constraints, respectively. Let *I* = [*c*, *d*] ⊂ *X* and *G* = {(*x*, *y*)∣*g*(*x*, *y*) ≥ 0, *x* ∈ *I*}. For the fixed *x* ∈ *I*, let $\bar{G}(I)$ denote the Pareto optimal solutions to the lower level problem; the induced set of problem (1) is denoted as $\Omega =\{(x,y)|(x,y)\in G,\;\;y\in \bar{G}(I)\}$. Note that the constraint *x* ∈ *I* is uncoupled from the lower level variable *y* ∈ *Y*. Particularly, if *m*_1_ = *m*_2_ = 2, we also call the BLMPP as bilevel biobjective programming problem (BLBOP). In the following, we shall focus on the BLBOP and assume that *m*_1_ = *m*_2_ = 2 and *n*_1_ = 1.

**Definition 1 .**

Let *K* be a closed pointed convex cone. A point $(\bar{x},\bar{y})\in G$ is called a Pareto optimal solution of the lower level problem with respect to *K* if $\left(f(\bar{x},\bar{y})-K)\cap f(G)=\{f(\bar{x},\bar{y})\right\}$.

**Definition 2 .**

For a fixed *x* ∈ *I*, if *y* is a Pareto optimal solution to the lower level problem, then (*x*, *y*) is a feasible solution to problem (1).

**Definition 3 .**

If (*x*^*∗*^, *y*^*∗*^) is a feasible solution to problem (1) and there are no (*x*, *y*) ∈ *Ω*, such that *F*(*x*, *y*)≺*F*(*x*^*∗*^, *y*^*∗*^), then (*x*^*∗*^, *y*^*∗*^) is a Pareto optimal solution to problem (1), where “≺” denotes Pareto preference.

**Definition 4 .**

If (*x*^*o*^, *y*^*o*^) is the optimistic solution for problem (1), then (*x*^*o*^, *y*^*o*^) is given by ${min}_{x,y}\{F(x,y)|y\in \bar{G}(x),\;x\in I\}$.

**Remark 5 .**

The optimistic solution to the BLBOP is the one that optimizes the leader's objective function over the set of efficient solutions to the follower, assuming that the follower has no preferences among the efficient solutions obtained for each leader's decision *x* or that the follower will choose the solution that most benefits the leader. In this paper, we only consider the optimistic BLBOP.

For problem (1), it is noted that a solution (*x*^*∗*^, *y*^*∗*^) is feasible for the upper level problem if and only if *y*^*∗*^ is an optimal solution for the lower level problem with *x* = *x*^*∗*^. In practice, we often make the approximate optimal solutions of the lower level problem as the optimal response feedback to the upper level problem, and this point of view is accepted usually. On the other hand, the authenticity of the lower level Pareto optimal solution is very important for the BLMP. If the obtained optimal Pareto solutions possess the fraudulence, it can lead to the failure to solve the whole problem. In this paper, we proposed the scalar approach for the lower level Pareto optimal solutions in order to improve the accuracy of the lower level Pareto optimal solutions.

## 3. Algorithm

### 3.1. The Scalar Approach for the Induced Set of BLBOP

In bilevel optimization, the constraint set of the upper level problem is given by the solution set of the lower level optimization problem. According to the Theorem 4.1 of the literature [55], the induced set *Ω* of problem (1) is equivalent to the Pareto optimal set of the multiobjective optimization problem

(2) miny f¯x,y=fx,yxs.t. x,y∈G x∈I.

Thus, to solve the induced set of BLBOP is transformed to solve the Pareto optimal solution set of problem (2). Inspired by the scalar approach adopted in [55], the approximation for the induced set of problem (1) can be solved by the following algorithm.

**Algorithm 1 .**

*Step 1*. Discretize the upper level variable.

*Step 1.1*. For one-dimension upper level decision variable, choose *θ* > 0 and discretize *I* = [*c*, *d*] by *x*_1_ = *c*, *x*_*k*_ = *x*_1_ + (*k* − 1)*θ*  (*k* = 2,3,…, *n* − 1), *x*_*n*_ = *d*.

*Step 1.2*. For *n*-dimension (*n* ≥ 2) upper level decision variable, choose *θ*_*i*_ > 0 and discretize *x* ∈ *I* = [*c*_1_, *d*_1_] × [*c*_2_, *d*_2_] × ⋯×[*c*_*n*_, *d*_*n*_] by {*x* ∈ *R*^*n*^∣*x*_*i*_ = *c*_*i*_ + *n*_*i*_*θ*_*i*_, *x*_*i*_ ≤ *d*_*i*_}.

*Step 2*. Initialize the loop variables *k* = 1 and predefine an accuracy measure *α* > 0.

*Step 3*. Execute the following steps for *x* = *x*^*k*^  (*k* = 1,2,…, *n*) and let $\tilde{a}=x^{k}$.

*Step 3.1*. Solve $f_{1}(x^{k},{\bar{y}}^{1})={min}_{x}\left\{f_{1}(x^{k},y)|(x^{k},y)\in G\right\}$ and $f_{2}(x^{k},{\bar{y}}^{2})={min}_{x}\left\{f_{2}(x^{k},y)|(x^{k},y)\in G\right\}$. Then, determine $a^{1}=(f_{1}(x^{k},{\bar{y}}^{1}),0)$ and $a^{M}=(f_{1}(x^{k},{\bar{y}}^{2}),0)$. Denote the approximate Pareto optimal solution set by $A^{0,k}=\left\{\left(x^{k},{\bar{y}}^{1}\right)\right\}$. Let $a^{2}=(f_{1}(x^{k},{\bar{y}}^{1})+\delta ,0{)}^{T}$ with a small *δ* > 0 and *t* = 2.

*Step 3.2*. If *a*_1_^*t*^ ≤ *a*_1_^*M*^, solve problem (2) using the scalarization method $SP(a^{l},r,\tilde{a})$ in [56] with $\tilde{a}=x^{k}$, *r* = (0,1)^*T*^ for the minimal solution *y*^*l*^. Set *A*^0,*k*^ = *A*^0,*k*^ ∪ {(*x*^*l*^, *y*^*k*^)}.

*Step 3.3*. Calculate the *M*, *N* according to Theorem 4.3 and determine $\bar{\lambda }$ according to (5.2) in [56]. Set $a^{t+1}=a^{t}+\bar{\lambda }\cdot (1,0{)}^{T}$ and let *t* = *t* + 1; go to Step 4.

*Step 4*. Output the approximation Pareto optimal solution set *A*^0^; that is, *A*^0^ = ⋃_*k*=1_^*n*^*A*^0,*k*^.

### 3.2. The Two-Stage ANN

If *θ* > 0 in Step 2 is small, then set *A*^0^ will consist of many points. Then, the determination of all nondominated points for the upper level problem can be very expensive. In this subsection, the two-stage ANN is presented for determining the Pareto optimal solution of the BLBOP on the induced set *A*^0^. The first stage is to map the vectors from *R*^*n*_1_+*n*_2_^ to *R*^*m*_1_^ and the second stage is to determine the Pareto optimal solutions of problem (1). The detail of each stage is described as follows.

The first stage of the ANN is a feed-forward artificial neural network (FFANN) which is composed by two subnetworks *A* and *B* with the same structure and neuron output function. For each subnetwork, the nodes are organized into two layers and the weighted arcs only link nodes in lower layers to nodes in higher layers. The first stage is to compute the objective function value for the upper lever.

The second stage is a quasineural artificial network; namely, the network has no connectivity weight and the output value can be computed directly by software. The input layer of the quasineural artificial network is the output layer of the first stage network. For the hidden layer, the input of and the output are defined by (3) and (4), respectively.

(3) Sp=fpxAi−fpxBjp=1,2,

(4) O1Sp=1if  Sp>0−1if  Sp≤0p=1,2.

For the output layer, the input of and the output are defined by (5) and (6), respectively.

(5) Sq=∑p=12O1Sp,

(6) O2Sq=1Sq=20Sq=0−1Sq=−2.

Based on the set *A*^0^ obtained by Algorithm 1, the first approximation Pareto optimal solutions of problem (1) can be achieved by the following algorithm.

**Algorithm 2 .**

*Step 1*. Discrete induced set *A*^0^, denote the discrete set by *A*_*s*_^0^, and let *s* = 0.

*Step 2*. Divide the set *A*_*s*_^0^ into two subsets *A*_*s*1_^0^ and *A*_*s*2_^0^ randomly; that is *A*_*s*_^0^ = *A*_*s*1_^0^ ∪ *A*_*s*2_^0^.

*Step 3*. Input *x*_*i*_ ∈ *A*_*s*1_^0^ and *x*_*j*_ ∈ *A*_*s*2_^0^ into subnetwork *A* and subnetwork *B*, respectively.

*Step 4*. Select Pareto optimal solutions.

*Step 4.1*. If *O*_2_(*S*_*q*_) = 1, then *A*_*s*1_^0^ = *A*_*s*1_^0^∖*x*_*i*_. Let *i* = *i* + 1, go to Step 4.

*Step 4.2*. If *O*_2_(*S*_*q*_) = −1, then *A*_*s*2_^0^ = *A*_*s*2_^0^∖*x*_*j*_. Let *j* = *j* + 1, go to Step 4.

*Step 4.3*. If *O*_2_(*S*_*q*_) = 0, then *x*_*i*_, *x*_*j*_ ∈ *A*_*s*+1_^0^. Go to Step 4.

*Step 5*. If *A*_*s*1_^0^ = *ϕ*, *A*_*s*2_^0^ ≠ *ϕ*, then (∀*x*_*j*_ ∈ *A*_*s*2_^0^) ⊂ *A*_*s*+1_^0^.

*Step 6*. If *A*_*s*1_^0^ ≠ *ϕ*, *A*_*s*2_^0^ = *ϕ*, then (∀*x*_*i*_ ∈ *A*_*s*1_^0^) ⊂ *A*_*s*+1_^0^.

*Step 7*. If the stopping criterion is met, then stop. Otherwise, go to Step 2.

In Step 2, the two subsets *A*_*s*1_^0^ and *A*_*s*2_^0^ have approximately equal number of feasible solutions. The output of Algorithm 2 is the first approximation Pareto optimal solution set of problem (1); we denote the set by *P*(*A*^0^).

### 3.3. The Algorithm for BLBOP

Based on Algorithms 1 and 2, as well as the refinement strategy used by the literature [9] which is employed in this paper, the algorithm for solving the BLBOP can be described as follows.

**Algorithm 3 .**

*Step 1*. Based on the induced set *A*^0^ obtained by Algorithm 1, we determine the approximation Pareto optimal solution set *P*(*A*^0^) of problem (1) by Algorithm 2.

*Step 2*. Let *i* = 0 and choose the distance *γ*^*i*^ > 0.

*Step 3*. For any point (*x*, *y*) ∈ *P*(*A*^0^), determine the refinement induced set around this point by the refinement strategy according to (5.4) in [55].

*Step 4*. Set $A^{i+1}=A^{i}\cup \left(\underset{\left(x,y\right)\in P\left(A^{0}\right)}{A_{\left(x,y\right)}^{i}}\right)$; update the approximation Pareto optimal solution set *A*^*i*+1^ by Algorithm 2.

*Step 5*. If approximation of solution set of problem (1) is sufficient, then stop. Otherwise set *i* = *i* + 1, choose *γ*^*i*^ > 0, and go to Step 3.

## 4. Results

In this section, we considered seven numerical examples and a practical problem to illustrate the feasibility of the proposed algorithm for problem (1). In order to evaluate the closeness between the obtained Pareto optimal front and the theoretical Pareto optimal front, as well as the diversity of the obtained Pareto optimal solutions along the theoretical Pareto optimal front, we adopted the following evaluation metrics.

### 4.1. Performance Evaluation Metrics

#### 4.1.1. Generational Distance (GD)

This metric used by Deb [65] is employed in this paper as a way of evaluating the closeness between the obtained Pareto optimal front and the theoretical Pareto optimal front. The GD metric denotes the average distance between the obtained Pareto optimal front and the theoretical Pareto optimal front:

(7) $$\begin{array}{c} GD=\frac{\sqrt{\sum_{i=1}^{n}d_{i}^{2}}}{n}, \end{array}$$

where *n* is the number of the obtained Pareto optimal solutions by the proposed algorithm and *d*_*i*_ is the Euclidean distance between each obtained Pareto optimal solution and the nearest member of the theoretical Pareto optimal set.

#### 4.1.2. Spacing (SP)

This metric is used to evaluate the diversity of the obtained Pareto optimal solutions by comparing the uniform distribution and the deviation of solutions as described by Deb [65]:

(8) $$\begin{array}{c} SP=\frac{\sum_{m=1}^{M}d_{m}^{e}+\sum_{i=1}^{n}{\left(\bar{d}-d_{i}\right)}^{2}}{\sum_{m=1}^{M}d_{m}^{e}+n\bar{d}}, \end{array}$$

where *d*_*i*_ = min_*j*_(|*F*_1_^*i*^(*x*, *y*) − *F*_1_^*j*^(*x*, *y*)| + |*F*_2_^*i*^(*x*, *y*) − *F*_2_^*j*^(*x*, *y*)|), *i*, *j* = 1,2,…, *n*, $\bar{d}$ is the mean of all *d*_*i*_, *d*_*m*_^*e*^ is the Euclidean distance between the extreme solutions in obtained Pareto optimal solution set and the theoretical Pareto optimal solution set on the *m*th objective, *M* is the number of the upper level objective function, and *n* is the number of the obtained solutions by the proposed algorithm.

All results presented in this paper have been obtained on a personal computer (CPU: AMD 2.80 GHz; RAM: 3.25 GB) using a c# implementation of the proposed algorithm.

### 4.2. Numerical Experiment

In this section, we will present seven BLBOPS to illustrate the proposed algorithm for the bilevel biobjective programming. Problem 1 and problem 2 are low-dimensional problems. Problems 3–6 are high-dimensional problems. For problem 7, the theoretical optimal front is unknown. In this paper, we refined every upper problem's feasible solutions three times and the obtained results are compared with the classical literature.

**Example 1 .**

Example 1 is taken from [57]. Here *x* ∈ *R*^1^, *y* ∈ *R*^2^. In this example, the parameters *γ*^0^, *γ*^1^, *γ*^2^ of Algorithm 3 are 0.23, 0.11, and 0.03.

(9) minx Fx,y=y1−x,y2s.t. G1y=1+y1+y2≥0miny fx,y=y1,y2s.t. g1x,y=x2−y12−y22≥0, −1≤y1,y2≤1,0≤x≤1.

**Figure 1:**
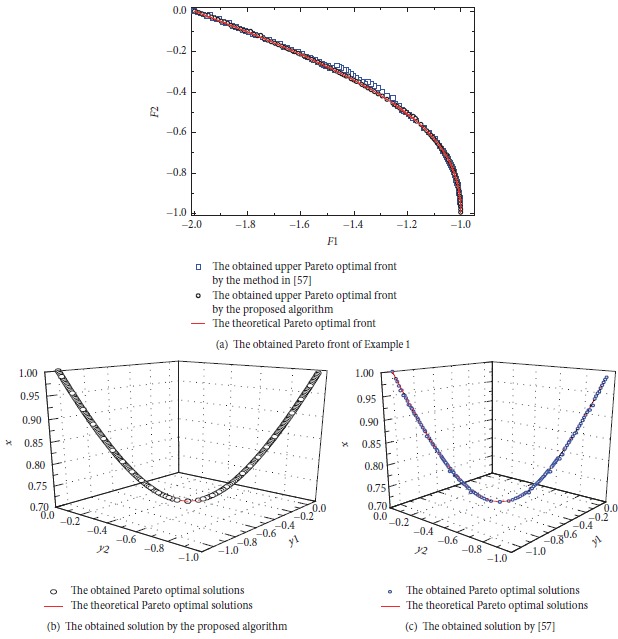
The obtained Pareto front and solutions of Example 1.

**Figure 2:**
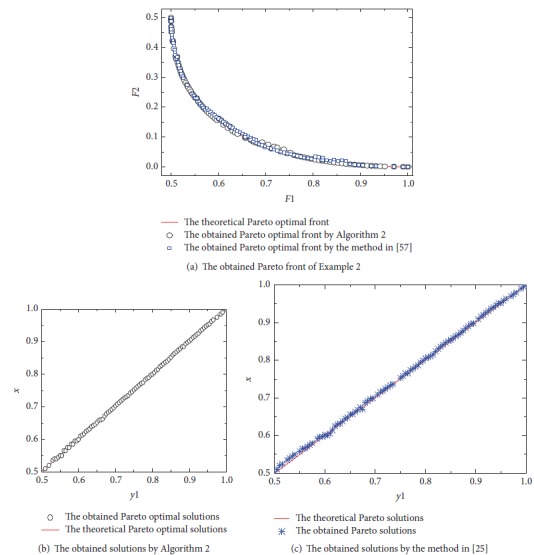
The obtained Pareto front and solutions of Example 2.

Figure 1(a) shows the obtained Pareto front by the proposed algorithm and the method in [57].  Table 1 shows the comparison results between the two algorithms considering the metrics previously described. It can be seen that the performance of the proposed algorithm is better with respect to the generational distance, although it places slightly below the method in [57] with respect to spacing. By looking at the obtained Pareto fronts, some nondominated vectors produced by the method in [57] are not part of the true Pareto front of the problem; however, the proposed algorithm is able to cover the full Pareto front.  Figure 2(b) shows that all the obtained solutions by the proposed algorithm, which follow the relationship; that is, *y*_1_ = −1 − *y*_2_, $y_{2}=-1/2\pm (1/4)\sqrt{8x^{2}-4}$, and $x\in (1/\sqrt{2},1)$. However, some solutions obtained by the method in [57] do not meet the relationship.

**Example 2 .**

Example 2 is taken from [57]. Here *x* ∈ *R*^1^, *y* ∈ *R*^2^. In this example, the parameters *γ*^0^, *γ*^1^, *γ*^2^ of Algorithm 3 are 0.3, 0.21, and 0.12.

(10) minx Fx,y=x2+y1−12+y22,x−12+y1−12+y22miny fx,y=y12+y22,y1−x2+y22 −1≤x,y1,y2≤2.

Figure 2(a) shows the obtained Pareto front by the proposed algorithm and the method in [57]. Obviously, both the proposed algorithm and the method in [57] almost have the same spacing. However, there are some areas of the Pareto optimal solution obtained by the method in [57] which is sparse. In Figures 2(a) and 2(b), it can be seen that the obtained solutions by the proposed algorithm almost follow the relationship; that is, *x* = *y*_1_,  *y*_1_ ∈ [0.5,1], *y*_2_ = 0. However, some areas of the solutions obtained by the method in [57] are sparse and some solutions do not meet the relationship.

**Example 3 .**

Example 3 is taken from [60]. Here *x* ∈ *R*^10^, *y* ∈ *R*^10^. In this example, the parameters *γ*^0^, *γ*^1^, *γ*^2^ of Algorithm 3 are 0.5, 0.36, and 0.12.

(11) minx Fx,y=1+r−cos⁡απx1+∑j=2Kxj−j−122+τ∑i=2Kyi−xi2−γcos⁡γπx12y1,1+r−sin⁡απx1+∑j=2Kxj−j−122+τ∑i=2Kyi−xi2−γsin⁡γπx12y1,miny fx,y=y12+∑i=2Kyi−xi2+∑i=2K101−cos⁡πkyi−xi,∑i=1Kyi−xi2+∑i=2K101−sin⁡πkyi−xis.t.  −K≤yi≤K,i=1,2,…,K; 1≤xi≤4, −K≤xj≤K,j=2,3,…,K, α=1, r=0.1, τ=1, γ=1, K=10.

**Figure 3:**
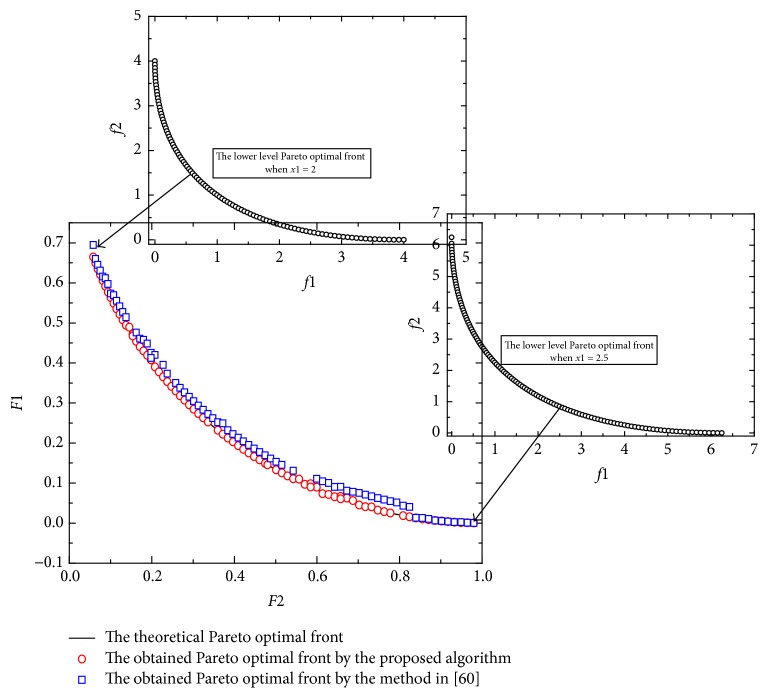
The obtained Pareto front of Example 3.

This problem is more difficult compared to the previous problems (Examples 1 and 2) because the lower level problem of this example has multimodalities, thereby making the lower level problem difficult in finding the upper level Pareto optimal front.  Figure 3 shows the graphical results produced by the method in [60] and the proposed algorithm in this paper. Tables 2 and 3 show the comparison of results between the two algorithms considering the metrics previously described. It can be seen that the performance of the proposed algorithm is the best with respect to the generational distance. By looking at the Pareto fronts of this test problem, some nondominated vectors produced by the method in [60] are not part of the true Pareto front and there are some areas are sparse. However, the proposed algorithm is able to cover the full Pareto optimal front. Furthermore, two obtained lower level Pareto optimal fronts are given when *x*_1_ = 2 and *x*_1_ = 2.5.

**Example 4 .**

Example 4 is taken from [60]. Here *x* ∈ *R*^10^, *y* ∈ *R*^10^. In this example, the parameters *γ*^0^, *γ*^1^, *γ*^2^ of Algorithm 3 are 0.6, 0.38, and 0.16.

(12) minx Fx,y=v1x1+∑j=2Kyj2+101−cos⁡πkyi+τ∑i=2Kyi−xi2−rcos⁡γπx12y1, v2x1+∑j=2Kyj2+101−cos⁡πkyi+τ∑i=2Kyi−xi2−rsin⁡γπx12y1,miny fx,y=y12+∑i=2Kyi−xi2,∑i=1Kiyi−xi2s.t.  −K≤yi≤K,i=1,2,…,K; 0.001≤xi≤4, −K≤xj≤K,j=2,3,…,K, α=1, r=0.25, τ=1, γ=4, K=10,

where

(13) v1x1=cos⁡0.2πx1+sin⁡0.2π0.02sin⁡5πx1for  0≤x1≤1x1−1−sin⁡0.2πfor  x1>1v2x1=−sin⁡0.2πx1+cos⁡0.2π0.02sin⁡5πx1for  0≤x1≤10.1x1−1−sin⁡0.2πfor  x1>1.

**Figure 4:**
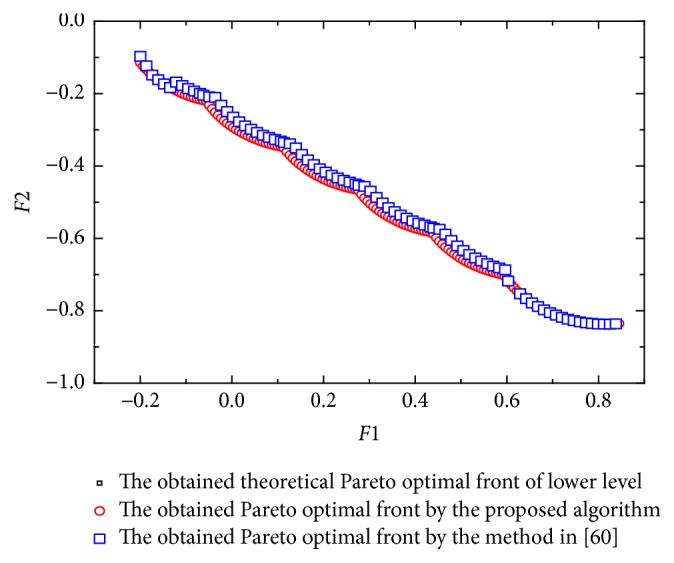
The obtained Pareto front of Example 4.

For Example 4, the upper level problem has multimodalities, thereby causing an algorithm difficulty in finding the upper level Pareto optimal front.  Figure 4 shows the obtained Pareto front by the method in [60] and the proposed algorithm. Tables 2 and 3 show the comparison of results among the three algorithms considering the metrics previously described. It can be seen that the performance of the proposed algorithm is better than the method in [60] with respect to the generational distance though they almost have the same performance with respect to the spacing.

**Example 5 .**

Example 5 is taken from [60]. Here *x* ∈ *R*^10^, *y* ∈ *R*^10^. In this example, the parameters *γ*^0^, *γ*^1^, *γ*^2^ of Algorithm 3 are 0.7, 0.49, and 0.23.

(14) minx Fx·y=x1+∑j=3Kxj−j22+τ∑i=3Kyi−xi2−cos⁡4 tan−1⁡x2−y2x1−y1, x2+∑j=3Kxj−j22+τ∑i=3Kyi−xi2−cos⁡4 tan−1⁡x2−y2x1−y1s.t. Gx=x2−1−x122≥0miny fx,y=y1+∑i=3Kyi−xi2,y2+∑i=3Kyi−xi2s.t. g1x,y=y1−x12+y2−x22≤r2 Rx1=0.1+0.15sin⁡2πx1−0.1, −K≤yi≤K,i=1,2,…,K, 0≤xi≤K,−K≤xj≤K,  i=1,2,…,K, τ=1, r=0.2, K=10.

**Figure 5:**
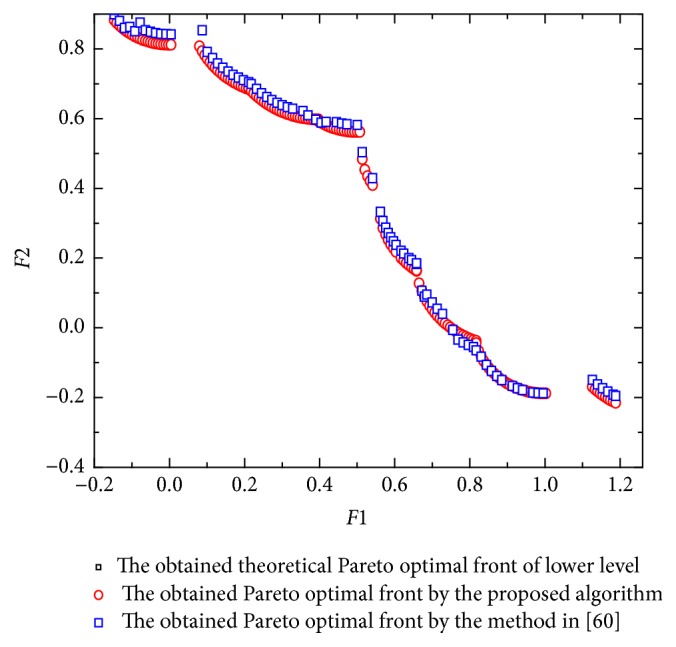
The obtained Pareto front of Example 5.

Figure 5 shows the obtained Pareto front of Example 5 by the method in [60] and the proposed algorithm. Tables 2 and 3 show the comparison of results between the two algorithms considering the metrics previously described. For this example, the graphical results again indicate that the method in [60] does not cover the full Pareto front. Since the nondominated vectors found by the method in [60] are clustered together, the spacing metric provides very good results. Graphically, it can be seen that the proposed algorithm is able to cover the entire Pareto front. It is also interesting to note that the Pareto optimal fronts for the upper level lie on constraint boundaries and every lower level Pareto optimal front has an unequal contribution to the upper level Pareto optimal front.

**Example 6 .**

Example 6 is taken from [60]. Here *x* ∈ *R*^1^, *y* ∈ *R*^9^. In this example, the parameters *γ*^0^, *γ*^1^, *γ*^2^ of Algorithm 3 are 0.5, 0.23, and 0.17.

(15) minx Fx,y=1−y11+∑j=2Kyj2x1,y11+∑j=2Kyj2x1s.t. G1x,y=−1−y1x1−12x1y1≤1,miny fx,y=1−y11+∑j=K+1K+Lyj2x1,y11+∑j=K+1K+Lyj2x1s.t. 1≤x1≤2, −1≤y1≤1, −K+L≤yj≤K+Lj=2,3,…,K+L,  K=5,  L=4.

**Figure 6:**
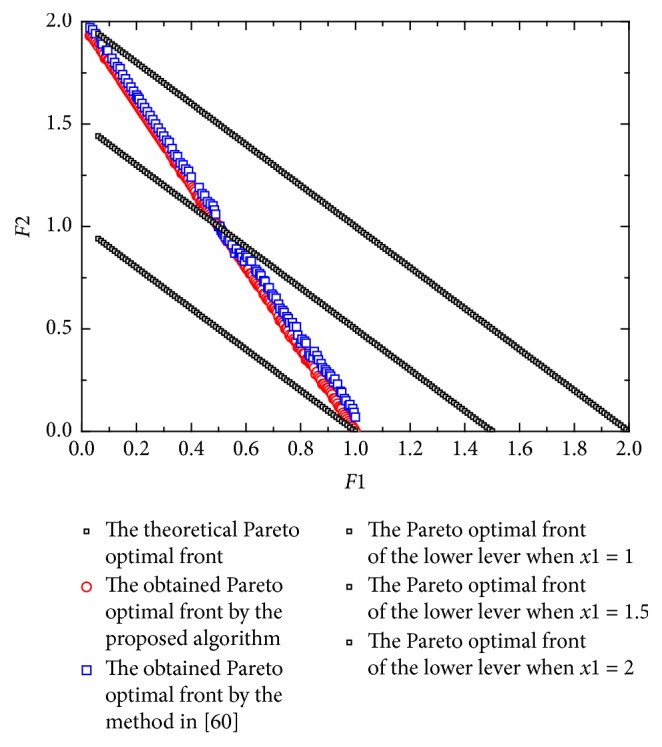
The obtained Pareto front of Example 6.

Figure 6 shows the obtained Pareto front of Example 6 by the method in [60] and the proposed algorithm. Tables 2 and 3 show the comparison of results between the two algorithms considering the metrics previously described. It can be seen that our algorithm and the method in [60] almost have the same performance of the spacing, but some nondominated vectors produced by the method in [60] are slightly off the true Pareto front. Moreover, three obtained lower level Pareto optimal fronts are given when *y*_1_ = 1, *y*_1_ = 1.5, and *y*_1_ = 2. It can be seen that only one Pareto optimal point from each participating lower level problem qualifies to be on the upper level Pareto optimal front.

**Example 7 .**

Example 7 is taken from [55]. Here *x* ∈ *R*^1^, *y* ∈ *R*^2^. In this example, the parameters *γ*^0^, *γ*^1^, *γ*^2^ of Algorithm 3 are 0.3, 0.16, and 0.07, respectively.

(16) minx Fx,y=x+y12+y22+sin2⁡x1+y,cos⁡y20.1+yexp⁡−y10.1+y2s.t. G1x,y=x−52−y1−0.52−y2−52≤16,miny fx,y=y1−22+y2−224+xy2+5−x216+sin⁡y210,y12+y2−62−2xy1−5−x280s.t. g1x,y=y12−y2≤0, g2x,y=5y12+y2−10≤0, g3x,y=y2−5−x6≤0, 0≤x≤10, 0≤y1,y2≤10.

**Figure 7:**
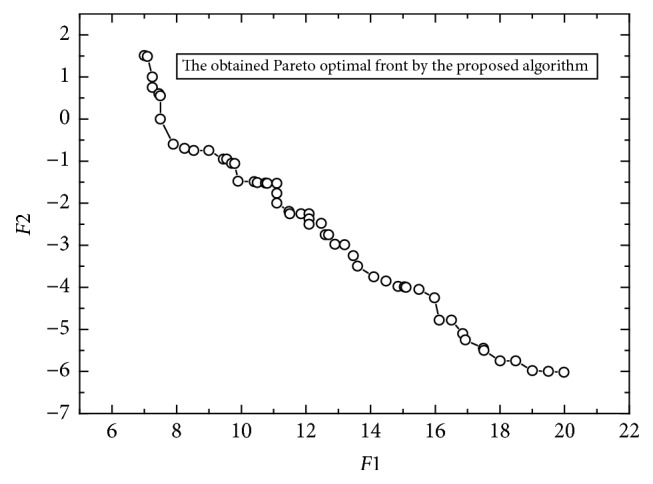
The obtained Pareto optimal solutions with *A*^3^.

Figure 7 shows the final archive solutions by the proposed algorithm. For this problem, the exact Pareto optimal front is not known, but the obtained Pareto optimal front after four approximations by the proposed algorithm is similar to that reported in the previous study [55].

### 4.3. Application of the Algorithm for a Practical Problem

**Figure 8:**
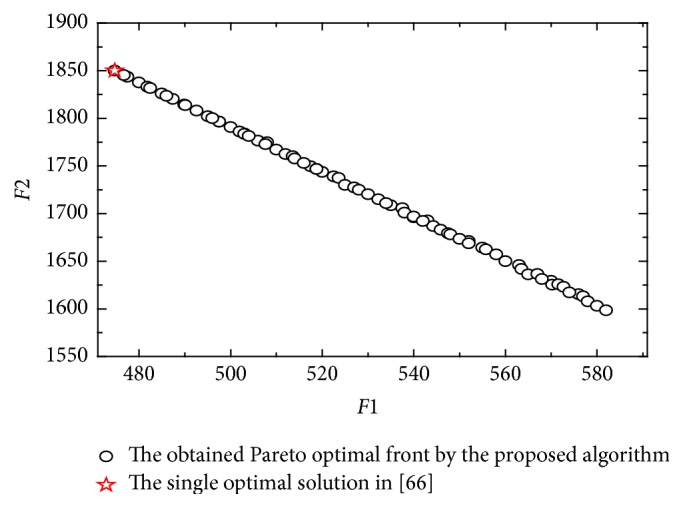
The obtained Pareto front of the practical problem.

In a company, the CEO's goal is usually to maximize net profits and quality of products, whereas a branch head's goal is to maximize its own profit and worker satisfaction. The problem involves uncertainty and is bilevel in nature, as a CEO's decision must take into account optimal decisions of branch heads. We present a deterministic version of the case study from [66] in (4).  Figure 8 shows the obtained Pareto optimal front of this practical problem by the proposed algorithm. Note that Zhang et al. [66] only obtained a single optimal solution *x* = (146.2955,28.9394) and *y* = (0,67.9318,0) which lies on the maximum of *F*_2_ using weighted sum method. In contrast, a set of Pareto optimal solutions is obtained by the proposed algorithm. However, the fact that the single optimal solution in [66] is included in the obtained Pareto optimal solutions illustrates the feasibility of proposed algorithm. In this problem, the parameters *γ*^0^, *γ*^1^, *γ*^2^ of Algorithm 3 are 0.2, 0.11, and 0.02.

(17) maxx Fx,y=x1+9x2+10y1+y2+3x3,9x1+2x2+2y1+7y2+4x3s.t. G1x,y=3x1+9x2+9y1+5y2+3y3≤1039 G2x,y=−4x1−x2+3y1−3y2+2y3≤94miny fx,y=4x1+6x2+7y1+4y2+8y3,6x1+4x2+8y1+7y2+4y3s.t. g1x,y=3x1−9x2−9y1−4y2≤61 g2x,y=5x1+9x2+10y1−y2−2y3≤924 g3x,y=3x1−3x2+y2+5y3≤420 x1,x2,y1,y2,y3≥0.

## 5. Conclusions

In this paper, a two-stage ANN based on scalarization method is presented for solving BLBOPs. Seven numerical examples and a practical problem are used to state the feasibility and efficiency of the proposed algorithm. The experimental results indicate that the obtained Pareto front by the proposed algorithm is very close to the theoretical Pareto optimal front, and the solutions are also distributed uniformly on entire range of the theoretical Pareto optimal front. The proposed algorithm is easy to implement, which provides another appealing method for further study on the general BLMPP.

## Figures and Tables

**Table 1:** Results of the Generation Distance (GD) and Spacing (SP) metrics for Examples 1 and 2.

Prob.	GD		SP	
	The method in [57]	The proposed method	The method in [57]	The proposed method
1	0.00216	0.00097	0.01135	0.01201
2	0.01013	0.00312	0.00203	0.00969

**Table 2:** Results of the Generation Distance (GD) metrics for Examples 3, 4, 5, and 6.

Prob.	The proposed algorithm	The method in [60]
3	0.00019	0.00768
4	0.00015	0.06391
5	0.00038	0.01677

6	0.00021	0.00652

**Table 3:** Results of the Spacing (SP) metrics for Examples 3, 4, 5, and 6.

Prob.	The proposed algorithm	The method in [60]
3	0.00076	0.00197
4	0.00273	0.00269
5	0.00299	0.01737

6	0.00130	0.00127
